# How Do Fungi Survive in the Sea and Respond to Climate Change?

**DOI:** 10.3390/jof8030291

**Published:** 2022-03-11

**Authors:** E. B. Gareth Jones, Sundari Ramakrishna, Sabaratnam Vikineswary, Diptosh Das, Ali H. Bahkali, Sheng-Yu Guo, Ka-Lai Pang

**Affiliations:** 1 Department of Botany and Microbiology, College of Science, King Saud University, P.O. Box 2455, Riyadh 11451, Saudi Arabia; torperadgj@gmail.com (E.B.G.J.); abahkali@ksu.edu.sa (A.H.B.); 2 Institute of Biological Sciences, Faculty of Science, University of Malaya, Kuala Lumpur 50603, Malaysia; sramakrishna@wwf.org.my (S.R.); viki@um.edu.my (S.V.); diptoshmycology@gmail.com (D.D.); 3 Institute of Marine Biology and Centre of Excellence for the Oceans, National Taiwan Ocean University, 2 Pei-Ning Road, Keelung 202301, Taiwan; alexsyguo@gmail.com

**Keywords:** ocean acidification, adaptation, deep sea, global warming, mangrove fungi, physiology, stress response, transcriptome, seawater

## Abstract

With the over 2000 marine fungi and fungal-like organisms documented so far, some have adapted fully to life in the sea, while some have the ability to tolerate environmental conditions in the marine milieu. These organisms have evolved various mechanisms for growth in the marine environment, especially against salinity gradients. This review highlights the response of marine fungi, fungal-like organisms and terrestrial fungi (for comparison) towards salinity variations in terms of their growth, spore germination, sporulation, physiology, and genetic adaptability. Marine, freshwater and terrestrial fungi and fungal-like organisms vary greatly in their response to salinity. Generally, terrestrial and freshwater fungi grow, germinate and sporulate better at lower salinities, while marine fungi do so over a wide range of salinities. Zoosporic fungal-like organisms are more sensitive to salinity than true fungi, especially Ascomycota and Basidiomycota. Labyrinthulomycota and marine Oomycota are more salinity tolerant than saprolegniaceous organisms in terms of growth and reproduction. Wide adaptability to saline conditions in marine or marine-related habitats requires mechanisms for maintaining accumulation of ions in the vacuoles, the exclusion of high levels of sodium chloride, the maintenance of turgor in the mycelium, optimal growth at alkaline pH, a broad temperature growth range from polar to tropical waters, and growth at depths and often under anoxic conditions, and these properties may allow marine fungi to positively respond to the challenges that climate change will bring. Other related topics will also be discussed in this article, such as the effect of salinity on secondary metabolite production by marine fungi, their evolution in the sea, and marine endophytes.

## 1. Introduction

The marine ecosystem is host to some 1900 fungi in 769 genera and 133 fungal-like organisms that have evolved for life in the sea (www.marinefungi.org, accessed on 15 December 2021) [1,2]. They include saprobes, parasites and endophytes, and are particularly common in mangroves (500 taxa, [3]) and salt marshes (486 taxa, [4]). This number of marine fungi is low in comparison to the number of terrestrial fungi, but marine fungi are predominantly saprobes and rely on the abundant organic matter available in coastal environments [5]. In the sea, a number of factors affects fungi growth, such as salinity, temperature [6], hydrostatic pressure in the deep-sea [7] and the anoxic conditions of the sediment [8]. Light may affect reproductive behavior of marine fungi [9]. Hydrocarbon [10,11] and plastic [12,13] pollution in the sea may also affect fungal growth behavior. Marine fungi/fungal-like organisms have evolved mechanisms for growth in the marine environment in response to salinity, and these are discussed in this article. In undertaking this review, it is important to also consider how terrestrial and freshwater fungi respond to saline conditions in estuaries and mangroves where there are great fluctuations in water salinity.

One of the first studies on the effect of salinity on growth of marine fungi was by Barghoorn and Linder [14], but have we made any progress in our understanding of the mechanisms that govern why fungi have been so successful in their colonization and adaptation to marine habitats? Not only do they tolerate high salinity conditions, but they also adapt to life in the sea. Too much time has been spent in trying to define what a marine fungus is, rather than understanding the different requirements of such a diverse fungal community. Perhaps we have to accept that not all marine fungi need to necessarily conform to the same physiological requirements in their response to salinity. The current definition of a marine fungus is "any fungus that is recovered repeatedly from marine habitats, because: (1) it is able to grow and/or sporulate (on substrata) in marine environments; (2) it forms symbiotic relationships with other marine organisms; or (3) it is shown to adapt and evolve at the genetic level or be metabolically active in marine environments" [15]. The perennial issue is for those fungi referred to as ‘marine-derived fungi’ often isolated in the search for novel bioactive compounds [15,16] and generally asexual morphs of genera such as *Aspergillus*, *Penicillium* and *Stanjemonium*. Endophytes/endozoans isolated from many plant and animal hosts, and those listed in metabarcoding studies are typical terrestrial taxa [17,18]. It is therefore appropriate to review what determines if a fungus is marine or is simply transient in the marine ecosystem [19].

The response of marine fungi to salinity gradients is of potential importance in terms of climate change with expected high temperatures, increased concentrations of salt, high hydrostatic pressures, and extreme pH. Knowledge of the underlying mechanisms for the adaptation of marine and terrestrial fungi to such events has implications for the biodiversity of marine habitats [20].

## 2. Growth of Terrestrial and Marine Fungi on Seawater Media

Early studies on marine fungi were dominated by surveys and descriptions of new taxa, their ability to decay wood, and salinity tolerance, especially the requirement for sodium concentrations, as in seawater. Thus, numerous studies have investigated the salinity tolerance of selected marine fungi [14,21,22,23,24,25,26,27,28,29,30,31,32,33,34], to mention but a few. Jones and Jennings [24] warned that a simple comparison of growth of fungi in distilled water and in seawater media does not give a complete picture of the growth of fungi under saline conditions. Here we comment on data derived over a range of salinities. A variety of responses have documented the growth of marine fungi, but generally mycelial growth occurred at all salinities with optimum growth varying from 10–50% seawater [24]. Two fungal-like organisms (*Althornia crouchi*, *Ostracoblabe implexa*) did not grow in distilled water or in 40% seawater [28]. Most terrestrial and freshwater fungi tested grew better at lower salinities with decreasing growth at higher salinities [22,28], however, a few showed optimum growth under fully saline conditions, especially asexual morphs like *Penicillium notatum* (100% seawater), and *Aspergillus flavus* (80% seawater). This tolerance of high salinities may account for why so many such genera are encountered in seawater column samples and in hydrothermal vents [1,35].

Subsequently, experiments were conducted to determine which elements in artificial seawater were tolerated by marine fungi in shake culture. There was no absolute requirement for sodium by *Paradendryphiella salina* (synonym *Dendryphiella salina*), but it enhanced growth at low salinities and inhibited growth at higher concentrations [36]. The addition of potassium to a basal medium produced the greatest growth. The bivalent cations magnesium, calcium and strontium inhibited dry weight at all concentrations, but removed sodium inhibition when added at low concentrations (25 M-equiv.). The same pattern was observed in other marine and terrestrial fungi, but varied from species to species. That study indicated that cation content in a medium is critical for the growth of fungi and the permeability of the fungal mycelium [36]. The sodium ion seems to be a key element affecting the growth of marine Chytridiomycota. A species of *Phlyctochytrium* produced the best growth at 237 mM sodium, and poor growth was observed at 0 mM or concentrations higher than 560 mM [37]. Mg(II) and Ca(II) were essential ions for growth of the same isolate *Phlyctochytrium* sp. [38].

Some fungi tolerate high concentrations of sodium chloride (NaCl), for example the asexual morph *Asteromyces cruciatus* tolerated concentrations of 2 M NaCl in liquid media with increased tolerance to 2.5 M NaCl with the addition of 0.05 M CaCl_2_. Jones and Ward [39] demonstrated that in media with high concentrations of sodium chloride, *Asteromyces cruciatus* produced septate conidia, rather than the normal unicellular conidia.

*Corollospora* is a species-rich genus, with most species occurring in association with sand and wood with a worldwide distribution [40,41,42]. Its arenicolous habit means species are exposed to great variations in salinity, especially during the intertidal period. Seventeen arenicolous fungi, ten of which are *Corollospora* strains, were grown on cornmeal agar in artificial seawater (100%) and at temperatures from 15–40 °C. There was little or no growth at 15 °C and their optimum temperatures are summarised in Table 1. Four *Corollospora* species were able to grow at 40 °C: *C. cinnamomea*, *C. colossa*, *C. maritima* and *C. pulchella*. The effect of salinity and temperature was also investigated with all the fungi exhibiting a positive response to varying salinities (Table 1). Group I are those that exhibited a higher salinity optimum, while Group II fungi had lower salinity optima of 60% and below at most temperatures tested. Four *Corollospora maritima* strains preferred a high salinity optimum for growth in 80–100% seawater.

In summary, studies have shown that growth of non-marine fungi was significantly less in seawater than in distilled water, while marine fungi were able to grow over a wide range of salinities. This reflects the ecological distribution of fungi as documented by Jones and Oliver [43] and Byrne and Jones [30,31]. Studies also underline that there is no specific requirement for high sodium concentrations in media for growth, but fungi can tolerate the cation ratios as in seawater.

## 3. Effect of Salinity on Spore Germination

Fungal spores are the usual means for the colonization of new substrates in the sea, and the physiological process of spore germination may be highly sensitive to various environmental factors, especially salinity. Few studies have explored this aspect of salinity tolerance. Byrne and Jones [30,31] examined the effect of salinity on the germination of ascospores and conidia of various marine and terrestrial fungi on agar media without added nutrients. Ascospores and conidia of the marine fungi *Asteromyces cruciatus*, *Corollospora maritima*, *Paradendryphiella salina* and *Lulwoana uniseptata* (synonym *Zalerion maritima*) germinated in distilled water and at all salinities of seawater. Doguet [44] reported similar results for the marine basidiomycete *Nia vibrissa.* Not all marine fungi germinate in distilled water, as reported by Meyers and Simms [25] for *Lindra thalassiae* and *Lulworthia floridana*. The terrestrial fungi *Chaetomium globosum*, *Mucor hiemalis* and *Penicillium notatum* did not germinate at higher salinities or showed reduced germination when grown at various salinities [30]. Borut and Johnson [45] demonstrated weak germination of *Aspergillus wentii*, *Penicillium janthinellum* and *Zygorhynchus moelleri* at low salinities, while the conidia of *Gliocladium fimbriatum*, *Paecilomyces puntonii* and *Trichoderma lignorum* germinated only in distilled water. To conclude, for terrestrial fungi percentage spore germination decreased with increasing salinity, while marine fungi exhibited a broad tolerance to salinity indicating an adaptation to the marine environment. Jennings [46] opined that the critical facility in the germination of spores/conidia of marine fungi is how the internal ionic environment is controlled as the spore germinates. Spores lack vacuoles for sequestering salt and the maintenance of turgor during germination [47]. Studies to elucidate the mechanism of spore germination in seawater remain relatively unexplored.

## 4. Fungal Sporulation with Salinity

**Figure 1:**
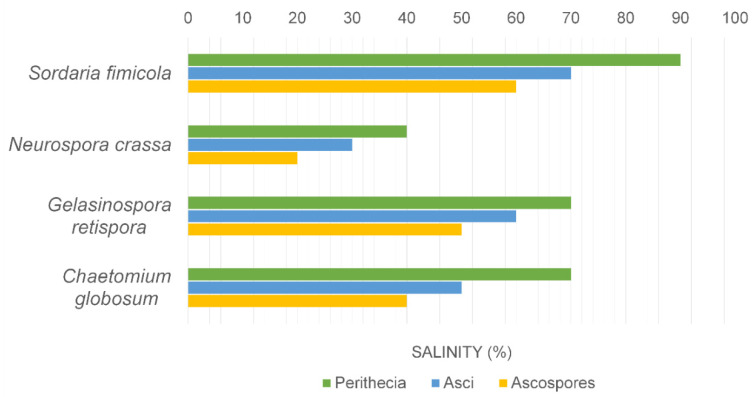
Effect of salinity on the production of perithecia, asci and ascospores of fungi.

The effects of seawater on the reproduction of marine and terrestrial fungi are aspects that have been little studied [28,48,49]. Marine ascomycetes such as *Halosphaeriopsis mediosetigera*, *Lulworthia floridana* and *Torpedospora radiata* sporulated on artificial media made up with seawater dilutions from 0–100%; however, the marine ascomycete *Lindra thalassiae* did not sporulate on freshwater media [28]. Jones et al. [28] showed that salinity had a pronounced effect on the sporulation of terrestrial fungi, for example *Chaetomium globosum*, *Gelasinospora retispora*, *Neurospora crassa* and *Sordaria fimicola*, with no viable ascospores formed above 60% seawater (Figure 1). Asci were formed at slightly higher salinities but were malformed or lacked spore delineation.

## 5. Fungal-like Organisms and Their Response to Saline Conditions

The fungal-like organisms of the Hyphochytriomycota, Oomycota and Labyrinthulomycota all have marine representatives, especially in mangrove habitats (www.marinefungi.org, accessed on 15 December 2021). Marine representatives of the orders Peronosporales, Pythiales and Saprolegniales (Oomycota) have been widely studied for their growth at various salinities and sodium chloride concentrations (Table 2 and Table 3). They are well adapted to the fluctuating salinities found in mangroves with the production of both asexual and sexual stages [50,51,52].

Few members of the Saprolegniaceae have been reported from marine habitats [56], and Harrison and Jones [57] questioned if this was due to their inability to tolerate high salinity levels. Using zoospore suspensions of 17 saprolegniaceous species, they investigated their ability to reproduce asexually at salinities of 0–40% seawater. Normal sporangial development occurred in freshwater, while in 10% seawater only nine produced normal zoospores: e.g., *Achlya bisexualis*, *Protoachyla paradoxa*, *Thraustyotheca clavata*, *Saprolegnia parasitica* and a *Saprolgenia* sp. In three *Isoachlya* spp. and *Achyla raceomsa,* some cytoplasmic cleavage occurred. In 20% seawater only small sporangial primordia were formed, with only *S. parasitica* and *T. clavata* producing zoospores. Formation of sexual reproduction was also investigated for the same taxa with most species forming oospheres, but in *I. toruloides* there was no cleavage of the oogonial cytoplasm. At 20% seawater only *P. paradoxa* produced mature oospheres, while at salinities above 20%, sexual reproduction was suppressed. Similar results were reported by Höhnk [58,59] for a *Saprolegnia* sp. when 7.09% salinity inhibited asexual reproduction, although excellent vegetative growth occurred. Clearly members of the Saprolegniales are not well adapted for survival at the higher salinities found in the ocean.

Species of the oomycetous genus *Halophytophthora* have wide salinity growth ranges. Isolates of *H. avicenniae* and *H. batemanensis* were able to grow at 4‰, 8‰, 16‰ and 32‰, although pH and incubation temperature had a combinatorial effect on growth [52]. The wide salinity growth ranges of *Halophytophthora* isolates suggest that they are well adapted to the salinity variation daily (high/low tide) and seasonally (summer/winter, rainy/dry seasons) in mangrove habitats.

## 6. Effect of Temperature and pH on Tolerance to Saline Conditions

In the marine environment, other abiotic factors such as water temperature and seawater pH also determine growth of fungi and subsequently their occurrence, in combination with salinity. Ritchie [21] first noticed an increase in salinity optimum of growth in *Phoma* sp. and *Pestalotia* sp. isolated from pine panels submerged in the sea with increasing incubation temperature. For example, the salinity optima of growth of *Phoma* sp. were 2%, 2.3%, 3.4% and 4.7% salt under 16 °C, 25 °C, 30 °C and 37 °C, respectively. This phenomenon, called ‘*Phoma* pattern’, was further confirmed in the marine fungi *Asteromyces cruciatus*, *Curvularia* sp., *Lignincola laevis*, *Nia vibrissa* and *Paradendryphiella salina* [60]. High temperature (35 °C) also caused an increase in salinity optima (45‰) coupled with maximal xylanase production in a salt marsh isolate of *Aureobasidium pullulans*, in addition to growth [61].

The ‘*Phoma* pattern’ was not observed in the Arctic wood-inhabiting marine fungus *Havispora longyearbyenensis*. Under 17‰ or 34‰ salinities, increase in temperature from 10 °C to 20 °C did not cause any effects on growth of *H. longyearbyenensis*; however, in freshwater, *H. longyearbyenensis* grew significantly better at a higher temperature (i.e., 20 °C) [62]. This is possibly a physiological adaptation of the fungus, when land ice melts during the summer months at Longyearbyen and dilutes the salinity of seawater.

Fungi isolated from sediment near the hydrothermal vent area of Kueishan Island, Taiwan had mixed responses to the combined effects of salinity, temperature and pH, and were categorized as (1) wide pH, salinity and temperature ranges, (2) salinity-dependent and temperature-sensitive, and (3) temperature-tolerant [63]. These fungi could grow in both freshwater and seawater (30‰), but growth was influenced by a medium pH and incubation temperature for some species. Some fungi such as *Aspergillus sydowii*, *Verticillium dahlia* and *Fondinomyces uranophilus* grew at 37 °C in the seawater medium, but not in the freshwater medium. The effects of pH on growth was mostly species-specific in relation to salinity.

## 7. Can Fungi Be Trained to Tolerate Saline Conditions?

This aspect of salinity tolerance has received little attention from marine mycologists. Byrne (unpublished data) grew *Chaetomium globosum* in seawater (SW/YGL) and distilled water (DW/YGL) yeast glucose liquid media for six generations for each of 10 days. At the final growth phase, they were tested on a range of salinities. The DW/YGL-grown material showed reduced growth with increased salinity, while the SW/YGL material produced increased growth with increased salinity. When the fungus was grown for 200 days on SW/YGL, optimum growth was at 80% seawater. Although there was mycelial adaptation/tolerance to seawater, no ascomata were produced. Under natural conditions, this inability to produce spores would lead to the adapted strain dying out.

This experiment was repeated for other fungi, including terrestrial: *Penicillium notatum* (16 days), freshwater: *Heliscus lugdunensis* (13 days), marine: *Corollospora maritima* (15 days), *Lulworthia* sp. (12 days) and *Paradendryphiella salina* (14 days) on agar media. Unlike *Ch. globosum*, *P. notatum* retained the ability to produce abundant fruiting structures. Marine taxa grown on DW/YGL showed no adaptation to freshwater conditions, but appeared to lose some growth capacity during prolonged exposure to these conditions, and this may be accounted for by the loss of ionic content in the media with time. Park et al. [64] demonstrated that *Aspergillus niger* exposed to 0.75% NaCl produced hyphae insensitive to this concentration of NaCl, but when increased stepwise was able to tolerate NaCl concentration up to 1.25%. Likewise, Sampangi-Ramaiah et al. [65] showed that a *Fusarium* sp., isolated as an endophyte from salt-adapted Pokkali rice, promoted the growth of a salt-sensitive rice variety. While some marine fungi show great plasticity to saline conditions, physiologically they do not require sodium for growth and at high concentrations it can inhibit growth.

## 8. Physiological Response to Salinity

This is a topic that has been widely researched by David Jennings and his students [46,66,67,68,69,70,71,72,73] who have carried out extensive studies on marine fungi and their ability to tolerate saline conditions in the marine environment. Do marine fungi require sodium chloride for growth and what are the mechanisms that control osmotic pressure within their mycelium? In an effort to understand these phenomena, a wide range of techniques have been applied to elucidate the mechanisms involved. Jennings [46] highlighted three physiological issues facing a fungus in the marine environment: seawater (1) has a relatively low water potential, (2) contains a relatively high concentration of ions, and (3) has an alkaline pH.

Studies with *Paradendryphiella salina* showed that sodium stimulates its growth at low salinities but is toxic at high concentrations [36]. This inhibition could be overcome by the addition of magnesium, calcium, strontium and barium (in order of effectiveness), and this was also demonstrated for other marine fungi [36]. The study also demonstrated that the key issue for the growth of the fungus was the permeability of the mycelium to potassium in a high salt medium. This study led to the exploration of a number of factors pertinent to understanding the mechanisms of salt tolerance in marine and other fungi [46]. These included plasma membrane permeability, accumulation of ions in the vacuoles, and the role of polyols in maintaining turgor in the mycelium.

In seawater, how does *Paradendryphiella salina* control the movement of nutrients and ions into the mycelium? It has already been shown that calcium and other bivalent cations play a role in the movement of potassium and exclusion of sodium from the mycelium. Jones and Jennings [36] proposed that there was a sodium pump for its exclusion from the mycelium. Galpin and Jennings [74] reported the involvement of ATPase in maintaining the K^+^/Na^+^ ratio within the mycelium of *P. salina*, and ATPase is required for the active transport of cations. When *P. salina* was grown at high salinity and pH, there was an increased activity of membrane-bound ATPase and this aids in good potassium/sodium balance in the cytoplasm [46]. Thus, ATPase is required for active transport of cations and fulfilled by glycolysis.

**Figure 2:**
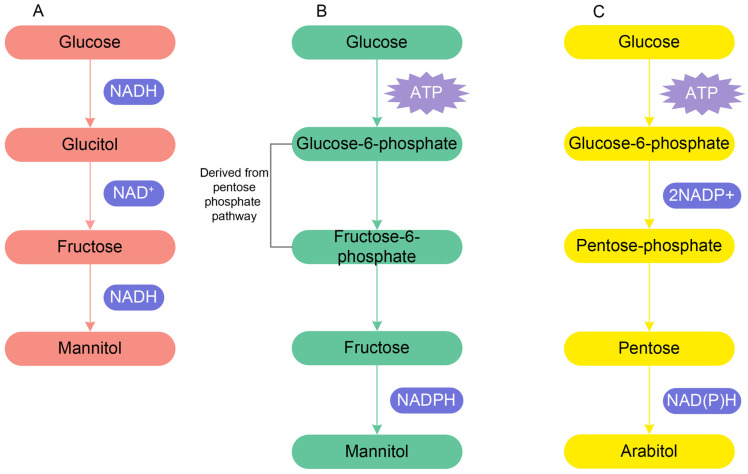
(**A**). Mannitol synthesis from glucose. (**B**). Mannitol synthesis from the hexose phosphate derived from the pentose phosphate pathway. (**C**). Arabitol synthesis from pentose sugar via the pentose phosphate pathway.

These studies continued by exploring the accumulation of ions in fungal vacuoles [68,72,75], plasma-membrane permeability [70,76] and the role of polyols in maintaining turgor in the marine fungal mycelium [73,75]. Ions contributed some 60% of the solute potential in the 48-h old mycelium of *P. salina* grown in the presence of high concentrations of sodium chloride, while polyols contributed 30% [75]. Jennings and Austin [72] showed the importance of mannitol and arabitol in maintaining the total in vivo carbohydrate content of mycelium of *P. salina* which is required for making optimum turgor for growth. Mannitol and arabitol synthesis within the hypha increased with increasing salinity because these sugar alcohols play the main role in maintaining osmotic pressure as well as correct differential water potential in the mycelium [66]. This was confirmed by Wethered et al. [75], who provided evidence that the polyol content of the mycelium increased with salinity. Holligan and Jennings [70] proposed two pathways of mannitol synthesis; one is directly from glucose entering the hyphae and another is from hexose phosphate derived from pentose phosphate pathway with ATPase hydrolysis (Figure 2A,B). Arabitol synthesis depends upon a stimulation of the pentose phosphate pathway and is derived from pentose sugar (xylulose and ribulose) via the pentose phosphate pathway (Figure 2C). Jennings [68] concluded that mannitol, arabitol, glycerol, and erythritol are the major polyols which are accumulated by mycelium, and variation in carbon sources, such as glucose and fructose, has an effect in the accumulation of various polyols.

In contrast to the mycelial fungi discussed above, there is evidence that zoosporic genera in the Thraustochytriales require sodium chloride for growth. Siegenthaler et al. [77,78] suggested that phosphate uptake in *Thraustochytrium roseum* required sodium chloride. Also, they demonstrated that the presence of the amino acid proline in their cells, as well as high levels of inorganic ions which contribute to the solute potential of the cells. Wethered and Jennings [79] noted that proline concentrations in cells increased with the increased salinity of the medium.

Norkrans [80] and Norkrans and Kylin [81] drew attention to marine occurring yeasts that are halotolerant with growth in the range of 0–24% sodium chloride. Gustafsson and Norkrans [82] and Adler and Gustafsson [83] reported that polyols accumulated in the marine occurring yeast *Debaryomyces hansenii* due to salt-stress, while Adler [84] showed that the accumulation of glycerol in *D. hansenii* played a role in osmoregulation.

Fungi in man-made salterns, soda lakes, coastal lagoons and the Dead Sea, tolerate very high environmental NaCl concentrations when compared to the marine fungi discussed here [85,86]. Larsen [85] characterised these fungi into four categories depending on tolerance to NaCl concentrations: non-tolerant up to 1%, slight 10%, moderate 20% and extreme 30% NaCl. These fungi too face similar physiological conditions to marine fungi, namely an external environment with relatively low water potential and high concentration of ions [87]. It is speculated that cation transporters prevent intracellular accumulation of Na^+^, which would be toxic but plays a role in maintaining the high K^+^/Na^+^ ratio required for growth in an environment with high salt content. The halophilic *Wallemia ichthyophaga* accumulates glycerol, while *Hortaea werneckii* also accumulates erythritol, arabitol and mannitol as solutes [88,89]. The same mechanism applies to the growth of marine fungi in seawater [46].

## 9. Salinity Effect on Production of Bioactive Compounds and Other Products

The total newly discovered marine fungal natural products stood at 4000 at the end of 2017, with additional ones in the intervening years [1]. There is little information available on metabolite production and salinity of the media used. Abbanat et al. [90] recorded that the yield of the anti-fungal agent 15G256ŷ increased from 3 to 400 mg/litre with the omission of seawater. This reflects that the optimum salinity for growth of many marine fungi is not in full strength seawater. Bugni and Ireland [91] commented that only two investigations examined the effect of varying salt concentrations in media and metabolite production [92,93]. These concluded that growth of the fungi increased with seawater concentration, but that maximum antimicrobial activity was in media with 25–50% seawater. Many studies lack information on the media used [94,95]. Lin et al. [96] and Toske et al. [97] specify that 30 g/L of NaCl and 100% seawater, respectively, were used for the fermentation medium. Janso et al. [98] explored the effect of media with 5, 10, 15 or 20% NaCl on the growth of *Penicillium dravuni* with the metabolites dityosphaeric acids A and B and carviolin produced in fermentation with 50% artificial seawater. Tepšič et al. [99] isolated *Aspergillus fumigatus* strains from salty soil, with salt concentration from 0.5–1 M NaCl and examined their potential to produce secondary metabolites. The production of secondary metabolites was much reduced and none of the mycotoxins investigated (verruculogen, fumitremorgins, fumagillin) were detected in media with water activity below 0.878 [99]. Overy et al. [100] also investigated the effect of salinity stress on the expression of secondary metabolite production in the terrestrial fungus *Aspergillus aculeatus*. Some metabolites increased or decreased in response to increasing osmolite, be it salt or glycerol concentrations. For example: aculene A (1) and B (2) decreased in yields when exposed to an increase in salinity. However, yields of the compounds CJ-15,183 and aspergillusol increased with increased salinity. Acu-dioxomphiline production increased with 50% seawater, but decreased in 100% seawater. Thus the response to increased salinity depended on the compound under consideration [100].

Gonçalves et al. [101] showed the marine isolate *Emericellopsis cladophorae* produced greater quantities of metabolites in salt-containing media and also more compounds such as ergocryptine, 2′-O-Galloylhyperin, (-)-Gallocatechin 3-gallate, and N-[1-(4-methoxy-6-oxopyran-2-yl)-2-methylbutyl]acetamide. However, in media lacking salt it produced other metabolites, thus having the potential to survive in both media.

## 10. Are There Genes That Control the Ability of Fungi to Survive in the Sea?

Whole genome sequences of the fungi *Hortaea werneckii*, *Wallemia ichthyophaga*, *Aureobasidium pullulans*, *A. subglaciale*, *A. melanogenum* and *A. namibiae* exhibit different levels of halotolerance, which are based on membrane transport systems that control physiological intracellular concentrations of alkali metal cations. Therefore, genes that control different K^+^ transporters are of particular interest. Genes that confer salt tolerance are HAK1 and HAL2 in *Debaryomyces hansenii* and their mode of action are detailed by Aggarwal et al. [102] and Prista et al. [103].

Table 1 lists the growth of various isolates of the arenicolous fungus *Corollospora maritima* at different temperatures with optimum growth at 80–100% seawater, while Velez et al. [42] showed their strain grew equally well in seawater and freshwater. Velez et al. [42] undertook a transcriptome analysis of *C. maritima* to determine its response to growth in seawater and freshwater. They showed that 103 genes were over-expressed in seawater, and 132 genes specifically up-regulated under freshwater. Genes detected may be responsible for cell wall biosynthesis and offer resistance to osmotic changes, namely GPI-anchored putative glucosidase and the aspartic-type endopeptidase. Further studies are required to elucidate the role of these genes. Pang et al. [63] summarized the genes that are possibly expressed during osmotic (salinity) stress from the literature, and they include arginine metabolism, aspartic-type endopeptidase, choline sulfatase, ergosterol, glutamate decarboxylase, glycerolipid metabolism (high osmolarity glycerol pathway, HOG), linoleic acid and pyrroline-5-carboxylate dehydrogenase.

Hagestad et al. [104], in discussing the genome of *Emericellopsis atlantica,* highlight the high G+C content that is linked to halotolerance in prokaryotes. They suggest that this may account for its adaptation to an environment with high salt content. Whole genomes for marine/halotoerant fungi are few in number (*Amylocarpus encephaloides*, *Aureobasidium pullulans*, *A. subglaciale*, *A. melanogenum*, *A. namibiae*, *Calycina marina*, *Corollospora maritima*, *Hortaea werneckii*, *Vercuulina enalia* and *Wallemia ichthyophaga*), and further efforts are required to sequence a wider range of taxa so as to understand their adaptability to the marine environment.

## 11. Ecological Occurrence of Marine Fungi

Many factors govern the geographical distribution of marine fungi, including the availability of substrates, osmotic response, oxygen availability, competition, and many others [105,106,107]. However, it is temperature and salinity that play a central role in their distribution. From the studies of Hughes [106,107,108], marine fungi have been grouped into: (1) Arctic-Antarctic or cold waters (*Lautosporopsis circumvestita*) [109], (2) temperate (*Lulworthia purpurea*) [110], (3) sub-tropical (not easily identified) and (4) tropical (*Antennospora quadricornuta*) [111]. Booth and Kenkel [105], based on ordination and concentration analysis, grouped lignicolous marine fungi into cool euryhalothermic, mixed, and warm euryhalothermic along the horizontal temperature gradient and salinity along the vertical axis. While temperature may be the dominant factor, salinity may play a significant role in estuaries and mangroves. Equally, seasonal wet/dry seasons in the tropics may underline the role of salinity [112]. Maria and Sridhar [113] highlighted the difference in mangrove occurrence of lignicolous fungi during the summer (dry) and monsoon (wet) seasons; fungal diversity was greatest during the monsoon period with terrestrial fungi dominant, while marine fungi dominated the mangrove community during the dry season.

Significant variation in the salinity of the water exists when considering estuaries and mangroves, yet few detailed studies have explored their fungal communities [43,114,115,116]. Jones and Oliver [43] found that the fungal communities were different in brackish and freshwater, with some common to both fresh and brackish water. Typical freshwater fungi, such as *Tricladium splendens* and *Lemonniera aquatica*, were found in the brackish zone of the river Towy (Carmarthenshire, Wales, UK) but not at the marine site. Shearer [114] found that species composition in the zone where salt and freshwater mixed (7.8–17.9‰) were significantly different in the Patuxent River (USA). Byrne and Jones [115] found marine fungi present at all sites in Yealm estuary (England) with little variation between the three sites. In a study of five sites along the Tutong River (Australia) and its tributary, with salinity ranging from freshwater, brackish to marine, the greatest number of fungi was at the freshwater site [116]. While some occurred only at the freshwater sites (*Annulatascus triseptatus*, *Torrentispora crassiparietis*) or marine (*Aniptodera megalospora*, *Savoryella lignicola*), others were found at all sites (*Cancellidium applanatum*, *Sungaiicola brachydesmiella)*.

In a different approach to the effect of salinity, Rojas-Jimenez et al. [117] investigated changes in fungal community composition along a salinity gradient in the Baltic Sea, based on 18S rRNA gene sequence analysis. At salinities below 8 PSU fungal communities similar to those from freshwater environments, namely the Chytridiomycota (especially the orders Rhizophydiales, Lobulomycetales, and Gromochytriales) were dominant. At salinities above 8 PSU, the Cryptomycota (Rozellomycota), Ascomycota and Basidiomycota were evident.

## 12. Did Marine Fungi Evolve in the Sea?

This is a topic that has been widely discussed in seeking an answer to the occurrence of marine fungi. Based on protein clock analyses by Heckman et al. [118], fungi emerged in oceans approximately 1 billion years ago during the Proterozoic era of the Precambrian with deep branches such as the Chytridiomycota [119]. During this period there was a lowering of the temperature and salinity, and increased dissolved oxygen during the Precambrian and Neoproterozoic [120,121,122]. So, this suggests that fungi initially occurred in the marine environment [119]. It is believed that the zoosporic Chytridiomycota is the sister group of the remaining phyla of non-flagellated fungi (Mucoromycota, Glomeromycota, Ascomycota and Basidiomycota), indicating a single loss of the flagellum coincident with a shift to land [120]. However, there is no compelling evidence to show that ancestral chytrids were marine [120]. Fungal evolution expanded during the Cretaceous with the dramatic increase in plants.

Many authors have indicated that the transition of fungi to the marine environment occurred many times and was not a one-off occurrence [123,124]. Vijaykrishna et al. [124] were of the opinion that fungi transitioned from freshwater as the number of ascomycete genera containing both terrestrial and freshwater species, along with marine taxa providing evidence for the migration of ascomycetes from land to the marine environment. The family Halosphaeriaceae has both freshwater and marine taxa, with the greater number of genera in the latter group. Interestingly, all genera in the Lulworthiales are known only from the marine environment (www.marinefungi.org, accessed on 15 December 2021). This topic is discussed in greater detail in Jones et al. [1].

**Figure 3:**
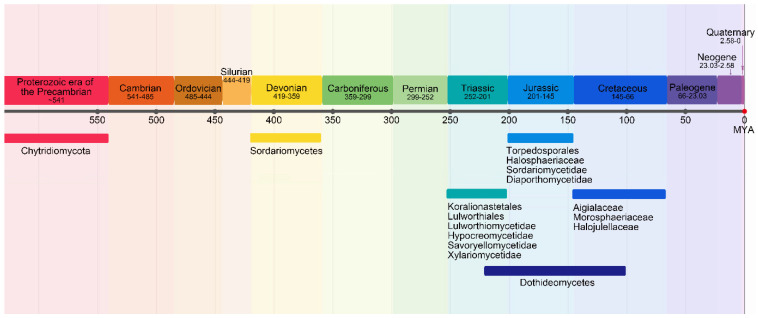
Time of evolution divergence of various groups of marine fungi.

With respect to different taxa, Spatafora et al. [125], and Campbell et al. [126] provided data that the Halosphaeriaceae are secondary marine ascomycetes, derived from terrestrial ancestors. From divergent time analysis, clearly taxa unique to the marine environments evolved at different times (Figure 3). Many marine fungi have adapted to an aquatic life by the passive release of spores, the development of ornate ascospore appendages to aide dispersal, and their attachment to selected host substrates. When considering the available divergence time estimates, Koralionastetales and Lulworthiales might be the earliest marine lineages among marine ascomycetes. Transition of the basidiomycetes to the marine environment resulted in the reduction in the size of the basidiocarp, and loss of ballistospory, e.g., as in *Halocyphina villosa* and *Nia vibrissa* [127]. Like marine Ascomycota, spores were often appendaged as in the *Nia vibrissa* and *Digitatispora* species [128,129].

## 13. Marine Fungi and Climate Change

Kumar et al. [130] opined on the ecology and evolution of marine fungi and their potential adaptation to climate change, but did not consider their physiology and tolerance of saline conditions. From the above review, marine fungi are unique with many characteristics that define their life in a saline environment. These include wide adaptability to saline conditions in mangroves/estuaries and salterns, mechanisms for maintaining accumulation of ions in the vacuoles, exclusion of high level of sodium chloride, maintaining turgor in the mycelium, optimal growth at alkaline pH, a broad temperature growth range from polar waters to higher temperatures in sand dunes/intertidal periods (0–40 °C), growth at depths and often under anoxic conditions [122]. With these features, marine fungi may well positively respond to the challenges that climate change will bring. Key amongst these will be an increase in CO_2_ levels, the predicted rise in temperatures, changes (dilution due to melting of the ice caps) in the salinity of seawater and rising sea-levels which will affect the distribution of sea grasses and mangrove/salt marsh plants [130].

1. An increase in CO_2_ levels will affect the acidity of seawater, which has implications for the growth of fungi with an alkaline pH requirement. Caldeira and Wickett [131] noted that seawater pH has dropped by 0.1 units, and may decrease by a further 0.7 units within the next three centuries. Krause et al. [132] carried out acidification experiments in microcosms with seawater from the Baltic Sea and recorded fungal abundance (as colony forming units). Their results suggested that even moderate acidification may lead to an increase in fungal abundance of almost an order of magnitude. Fungi present in this study were not identified, and so further studies are required to better understand the issue of ocean acidification on fungal communities.

2. Marine fungi have a broad tolerance to variation in salinity in terms of mycelial growth, spore germination and sporulation, and therefore should adapt to changes in oceanic salinity (see Figure 1).

3. Marine fungi appear to tolerate a wide range in seawater temperature (See Table 1). Although marine fungi are worldwide in distribution, certain taxa may be restricted geographically to the tropics, subtropics, temperate or polar waters [106,107,111,133,134]. However, there is little overlap in fungal species from tropical and temperate regions [135]. Consequently, many marine fungi in temperate regions will have to adapt to increased temperature. Pang et al. [63] have shown that an *Aspergillus terreus* strain isolated from a shallow hydrothermal vent was able to grow at 45 °C, pH 3, and 30% salinity.

## 14. Unresolved Issues: Endophytes

Many issues remain unresolved, such as, what form do the endophytes/endozoans of fungi in marine plants and animals take [136,137,138]. Are endophytes of marine plants well adapted physiologically to the marine milieu? Exploration of fungi isolated from substrates other than woody tissue has revealed an exciting and much greater diversity of taxa: the seagrass *Posidonia oceanica* [139,140,141], macroalgae *Asparagopsis taxiformis* [142], *Flabellia petiolata*, *Padina pavonica* [141], *Pterocladiella capillacea* [143], various macroalgal species [101,144], marine sponges *Dysidea fragilis*, *Pachymatisma johnstonia*, *Sycon ciliatum*, *Grantia compressa* [145,146], and sea cucumber *Holothuria poli* [147] (Table 4). Many of the taxa were isolated as pale to dark brown chlamydospores, e.g., *Corollospora*, *Neodevriesia* and *Paralulworthia* species [141,144]. No sexual stages were observed, but in what form do they occur within their hosts and how did they colonise their hosts? Have any attempts been made to induce sporulation in these endophytes/endozoans? Also, many taxa belonging to the Basidiomycota were isolated (e.g., *Bjerkandera adusta*, *Irpex lacteus*, *Psathyrella candolleana*), where did the inoculum come from and how were the hosts infected? Similarly, many Basidiomycota have been reported from deep sea sediments (e.g., *Pleurotus pulmonarius*, *Trametes versicolor*), so where did they come from?

In essence few studies have been conducted as to the form fungi take within marine plant/alga hosts [152,153]. Generally, the hyphae of endophytes occur as intracellular infections, rarely penetrating the host plant cells, e.g., *Rhabdocline parkeri* hyphae in Douglas fir (*Pseudotsuga taxifolia*) needles, *Phyllosticta abietis* in Giant fir (*Abies grandis*) needles while *Stagonospora innumerosa* grew within the epidermal cell of *Juncus effusus* var. *pacificus* [154]. Aletaha et al. [153] reported hyphae intercellularly within roots of various Chenopodiaceae species, and in many cases the hyphae were melanized.

Stanley [152] examined marine fungi growing on various algal hosts (*Lautitia danica* on *Chondrus crispus*, *Mycaureola* on *Dilsea carnosa*, *Mycosphaerella ascophylli* on *Ascophyllum nodosum*), especially their distribution within their internal tissues at the light, scanning and transmissions electron microscope, and reported both inter- and intra-cellular penetration. In the three algae generally, hyphae were distributed throughout the algal tissues with hyphal sheaths surrounding the filaments. In *D. carnosa*, penetration of algal cells was initially by fine bifurcate penetration hyphae. Only in this alga was there breakdown in algal cell walls and contents, with damage to chloroplasts and dissolution of Floridean starch grains, resulting in necrotic lesions surrounded by a ring of basidiomata [128,138].

Panno et al. [139] showed that a number of taxa isolated from *Posidonia oceanica* were able to detoxify lignocellulose residues in the presence of high salt concentrations, confirming that marine endophytes tolerate conditions in the marine milieu. Many studies confirm that endophytes isolated from completely submerged plants are able to grow at salinities as in seawater [101,142,145].

## 15. Conclusions

Both terrestrial and marine fungi vary greatly in their response to changes in salinity. Zoosporic fungi are more sensitive to seawater concentrations than members of the Ascomycota and Basidiomycota. Freshwater saprolegniaceous organisms rarely occur at salinities above 20% seawater and fail to produce zoospores or sexual reproductive organs at these salinities. Marine members of the Peronosporales, Pythiales and Saprolegniales (Oomycota) and Labyrinthulomycota may be considered stenohaline or tolerant of a broad range of salinities found, for example, in estuaries or mangrove habitats, producing both asexual and sexual reproductive structures. Thus, for zoosporic organisms, the response to salinity is well defined, and the definition to “occur, grow and sporulate” in the marine environment is appropriate.

In this article we have reviewed a number of factors that may play a role in the growth of fungi in a marine environment. Physiologically, fungi in seawater must maintain mycelial turgor and the absorption of nutrients, but it would appear that these apply to a wide range of non-marine fungi as well. The ability of fungi to reproduce either sexually and asexually in seawater is crucial to their long-term survival, as the inability of terrestrial Ascomycota and saprolegniaceous organisms to sporulate under saline conditions testifies. Many asexual morphs, like the so-called “marine-derived fungi” *Aspergillus*, *Penicillium* and *Cladosporium*, have wide tolerance to environmental conditions, hence their ability to also withstand life in the sea.

## Figures and Tables

**Table 1:** The optimum salinities (% seawater) for the growth of marine fungi at each temperature investigated. NB: All data used in this table were taken from the linear part of the growth curve. -: no growth; nt: not tested.

Fungi	Temperature (°C)						Culture in Days
	5 °C	15 °C	25 °C	30 °C	35 °C	40 °C	
**Group I (high salinity optima)**							
*Arenariomyces trifucatus*	-	80	80	80	100	-	35
*Corollospora besarispora*	-	80	80	60	80	-	42
*Corollospora cinnamomea*	-	20	100	100	100	100	12
*Corollospora colossa*	-	40	100	80	100	100	30
*Corollospora maritima* CM1	-	-	100	80	80	100	9
*Corollospora maritima* PP4169	-	80	100	80	-	nt	13
*Corollospora maritima* PP5089	-	20	100	80	100	nt	13
*Corollospora maritima* PP5197	-	100	100	100	-	nt	49
*Corollospora novofusca*	-	20	40	100	100	-	20
*Corollospora pulchella*	-	80	60	60	80	100	15
*Corollospora gracilis*	-	100	80	80	100	nt	13
*Savoryella appendiculata*	-	100	60	80	80	-	48
*Torpedospora radiata*	-	80	60	60	200	-	8
*Asteromyces cruciatus*	80	80	100	100	-	nt	7
*Lulworthia crassa*	-	80	80	60	-	-	16
**Group II (low salinity optima)**							
*Carbosphaerella leptosphaerioides*	-	60	40	60	60	-	28
*Corollospora lacera*	20	0	20	100	-	-	25

**Table 2:** Growth of marine Oomycota and Labyrinthulomycota in various concentrations of sodium chloride.

Species	Growth Optimum	Remark	Reference
**Oomycota**			
*Haliphthoros milfordensis*	2.5–3.0%	Little or no growth at 0–1.5%	[53]
**Labyrinthulomycota**			
*Oblongichytrium multirudimentale*	2.5–3.0%	No growth at 0% or above 5.0%	[54]
*Thraustochytrium motivum*	2.5–3.0%	No growth at 0% or above 5.0%	[54]
*Thraustochytrium roseum*	2.5–5.0%	Little or no growth at 0.1–0.5%	[54]
*Schizochytrium aggregatum*	2.5–3.0%	Little or no growth at 0.5–1.0%	[55]

**Table 3:** Growth and reproduction of marine Oomycota at various concentration of seawater (‰) (adapted from [51]).

Species	Growth Optimum	Sporulation Optimum
*Halophytophthora avicennae*	10–20 (up to 60)	10–30 (none above 35)
*Halophytophthora vesicula*	15–25 (up to 60)	10–15 (none above 35)
*Phytopythium kandeliae*	10–35 (none above 35)	15–35 (none above 35)
*Salispina lobata*	20–40 (none above 40)	30–40 (none above 40)
*Salisapilia masteri*	20–35 (up to 60)	30 (none above 40)

**Table 4:** Endophytes/endozoans isolated from different substrates in marine habitats.

Hosts	Dominant (Most Speciose) Fungal Taxa	Reference
*Fucus*, *Ulva*, *Enteromorpha* (macroalgae)	*Emericellopsis*, *Parasarocladium*	[101]
*Posidonia oceanica* (seagrass)	*Penicillium*, *Cladosporium*, *Acremonium*	[139]
*Posidonia oceanica* (seagrass)	Dothideomycetes (Pleosporales and Capnodiales) and Leotiomycetes (Helotiales)	[140]
*Posidonia oceanica* (seagrass), *Flabellia petiolata*, *Padina pavonica* (macroalgae)	*Penicillium*, *Cladosporium*	[141]
*Pterocladiella capillacea* (macroalgae)	*Aspergillus*, *Cladosporium*, *Penicillium*, *Rhodosporidium*	[143]
*Dysidea fragilis*, *Pachymatisma johnstonia*, *Sycon ciliatum* (marine sponges)	*Cladosporium*, *Penicillium*	[145]
*Grantia compressa* (marine sponge)	*Cladosporium*, *Penicillium*	[146]
*Holothuria poli* (sea cucumber)	*Aspergillus*, *Penicillium*	[147]
*Carcinus maenas* (marine crab)	*Ophiocordyceps*	[148]
*Suberites zeteki*, *Mycale armata* (marine sponges)	Malasseziales	[149]
*Zostera muelleri* (seagrass)	Pleosporales, *Wallemia ichthyophaga*	[150]
*Cymbastela concentrica*, *Scopalina* sp., *Tedania anhelans* (marine sponges)	*Epicoccum*, *Cladosporium*	[151]

## Data Availability

Not applicable.
